# Endogenous progesterone blurs frontostriatal representations of emotion control in healthy young women

**DOI:** 10.1017/S0033291726105315

**Published:** 2026-09-03

**Authors:** Macià Buades-Rotger, Rosamunde Hendricks, Beatrice Roncarati, Marta Calderón-García, Kaja Bergmann, Francisco José Pérez-Cano

**Affiliations:** 1 Department of Clinical Psychology and Psychobiology, Faculty of Psychology, University of Barcelona, Barcelona, Spain; 2 Institute of Neurosciences (UBneuro), University of Barcelona, Barcelona, Spain; 3 Department of Psychobiology and Methodology of Health Sciences, Autonomous University of Barcelona, Barcelona, Spain; 4 Hospital del Mar Research Institute, Barcelona, Spain; 5 School of Psychology, University of New South Wales, Sydney, New South Wales, Australia; 6 Physiology Section, Department of Biochemistry and Physiology, Faculty of Pharmacy and Food Sciences, University of Barcelona, Barcelona, Spain; 7 Institute of Research in Nutrition and Food Safety (INSA), University of Barcelona, 08921 Santa Coloma de Gramenet, Spain

**Keywords:** affect, emotion, estradiol, mood, multivoxel pattern analysis (MVPA), progesterone

## Abstract

**Background:**

Fluctuations in ovarian hormones are associated with changes in mood and emotional well-being. However, the neural mechanisms linking endocrine states and affective regulation remain poorly understood. We aimed to investigate how endogenous estradiol and progesterone modulate brain representations underlying emotion–action control in healthy young women.

**Methods:**

A total of 116 naturally cycling women performed an approach–avoidance task with emotional facial expressions during functional magnetic resonance imaging. Multivoxel pattern analyses were used to examine the neural encoding of emotion–action contingencies, i.e., classifying task conditions from multivariate activation patterns. We then tested whether salivary concentrations of estradiol and progesterone were associated with classification accuracy in various brain regions.

**Results:**

Participants responded more slowly and less accurately when approaching angry faces or avoiding happy ones, reflecting control over automatic emotional tendencies. Multivoxel decoding revealed that task conditions could be reliably distinguished from activation patterns in the lateral frontal pole (FPl), striatum, central insula, dorsal and ventral cerebellum, primary visual cortex, and periaqueductal gray. Importantly, higher progesterone levels were correlated with reduced classification accuracy in the FPl (*r*=-.26, *p*=.006) and striatum (*r*=-.26, *p*=.007), and lower FPl decodability was linked to poorer mood during the preceding week (*r*=-.25, *p*=.011). Moreover, decoding accuracy in the FPl fully mediated the indirect association between progesterone and worse mood (*R* ^2^=7.1%).

**Conclusions:**

These findings suggest that progesterone-related alterations in frontostriatal affective representations may contribute to mood variation.

## Introduction

By default, humans pursue reward signals and avoid punishment ones, i.e., *congruent* approach–avoidance behavior. In contrast, most individuals show difficulties when they must approach aversive stimuli and avoid positive ones, i.e., an *incongruent* emotion–action mapping (Roelofs, Bramson, & Toni, 2023). Neuroimaging studies have demonstrated that the control of approach–avoidance behavior depends on the lateral frontal pole (FPl), which modulates amygdala activation (Bramson et al., 2020; Lapate, Ballard, Heckner, & D’Esposito, 2022). Lower FPl activity has been linked not only with anxiety (Bramson et al., 2023; Zhuang et al., 2025) and posttraumatic stress symptoms (Kaldewaij et al., 2021) but also with psychopathy (Volman et al., 2016) and aggressiveness (Kaldewaij et al., 2019). Thus, FPl-dependent arbitration of approach–avoidance conflict is a promising transdiagnostic marker for both internalizing and externalizing psychopathology. In this study, we interrogated whether ovarian hormones such as estradiol and progesterone are related to the neural control of automatic action tendencies and whether this can predict mood in healthy young women.

The steroid hormone testosterone is associated with weakened FPl-dependent emotion control (Kaldewaij et al., 2019; Volman et al., 2016). Testosterone induces this imbalance by potentiating threat reactivity in the amygdala (Radke et al., 2015) upon binding to the many androgen receptors present in this structure, particularly its medial nucleus (Simerly, Swanson, Chang, & Muramatsu, 1990). Much less is known, however, regarding the role of estradiol and progesterone in the neural regulation of affect. Estradiol stimulates the growth of dopamine and serotonin receptors in reward-processing brain structures such as the ventral striatum (Fink et al., 1996). Estradiol has also been associated with larger reward prediction error responses in the ventral striatum, which indirectly indexes dopaminergic signaling (Bayer et al., 2019). This may partly underlie the improvements in mood seen in women who undergo hormone replacement therapy with estrogen (Sharma et al., 2023) or the robust increases in assertiveness reported in periovulatory women (Blake, Bastian, O’Dean, & Denson, 2017; Blake, McCartney, & Arslan, 2022), among other effects. Critically, endogenous estradiol is also associated with greater activity in emotion-regulatory brain areas such as the dorsolateral prefrontal cortex or the anterior cingulate cortex (Chung et al., 2019; Sharma et al., 2021), as well as increased hippocampal activity during successful retrieval of emotional stimuli (Bayer et al., 2018) and spatial navigation (Pletzer, Harris, Scheuringer, & Hidalgo-Lopez, 2019). Exogenously administered estradiol can similarly enhance prefrontal activation (Rehbein et al., 2021) and frontoparietal connectivity (Derntl et al., 2024) during emotion regulation. It should be noted that estradiol may not be univocally beneficial for mood, as its enhancing effect on dopaminergic signaling can also promote maladaptive risk-taking (Peters et al., 2025). Still, the premenstrual drop in estradiol levels has been consistently linked with anhedonia and impaired executive function (Peters et al., 2025).

In contrast to estradiol, progesterone has been predominantly suggested to negatively impact affect. Although allopregnanolone (progesterone’s main metabolite) is linked with better mood during pregnancy (Paul, Pinna, & Guidotti, 2020), premenstrual dysphoria coincides with – but is not necessarily caused by – the monthly progesterone peak (Sundström-Poromaa, Comasco, Sumner, & Luders, 2020). Concordantly, women with premenstrual dysphoric disorder show greater amygdala and insula reactivity to emotional stimuli, particularly in the luteal menstrual cycle phase (Stiernman et al., 2025). Exogenous progesterone administration likewise heightens amygdala reactivity to emotional stimuli (van Wingen et al., 2007) and increases irritability among women with premenstrual dysphoria (Wei et al., 2025). Complementing these findings, pharmacologically maintaining progesterone levels low and stable facilitates the prefrontal regulation of aggression in women with premenstrual dysphoria (Kaltsouni et al., 2021). These effects are altogether consistent with studies showing that exogenous progesterone impairs affective processing (Derntl et al., 2008; Guapo et al., 2009), though recent evidence has questioned a direct association between endogenous progesterone and emotion recognition (Pletzer & Noachtar, 2023; Rafiee et al., 2023). Endogenous progesterone has however been reported to increase frontostriatal activation during spatial navigation (Pletzer et al., 2019) as well as thalamic reactivity to negative stimuli in a working memory task (Sharma et al., 2021).

In sum, estradiol generally improves mood by potentiating prefrontal and hippocampal activation along with dopaminergic reward signals in the striatum; progesterone, on the other hand, may worsen affect by sensitizing subcortical structures to threat signals as well as by diminishing top-down prefrontal control (Peters et al., 2025). Nonetheless, most neuroimaging studies on the topic have relied on univariate measures of regional activity or connectivity, inspecting whether individual brain regions become more or less active or synchronized with one another. Consequently, it remains unclear how ovarian hormones shape distributed neural representations underlying emotion regulation, i.e., the multivariate configuration of activity patterns across populations of voxels that encode affective states and action tendencies (Lapate et al., 2022; Weaverdyck, Lieberman, & Parkinson, 2020). Characterizing these representations may provide important insights into the neural pathways linking ovarian hormones to variability in emotional functioning. Moreover, previous work has mostly employed relatively small samples, which often yield imprecise effect sizes (DeYoung et al., 2025). In addition, commonly employed tasks often fail to engage the FPl and thus miss out on its potentially crucial contribution to emotion control (Bramson et al., 2020; Koch, Mars, Toni, & Roelofs, 2018). Here, we adopted a multivariate approach in a sample of naturally cycling healthy young women (*n* = 116) to examine whether estradiol and progesterone are associated with neural representations of emotion control, operationalized as the decodability of task conditions from distributed patterns of brain activity, and whether variation in these representations relates to mood. In so doing, we follow recently proposed guidelines to maximize the reliability of brain–behavior associations (DeYoung et al., 2025), namely, (a) theoretical matching between tasks and constructs, as we used an approach–avoidance task (AAT) that robustly recruits the FPl and predicts both internalizing and internalizing symptoms across the clinical and subclinical spectra (Bramson et al., 2023; Buades-Rotger et al., 2021; Kaldewaij et al., 2019, 2021); (b) individualized measures, as we extracted maximal effects for each participant from their own gray matter masks to best capture interindividual variability; and (c) cross-validated multivariate approaches, as we implemented a multivoxel decoding analysis where data from odd trials was used to predict unseen activation patterns in even trials. Indeed, multivariate methods leverage the high-dimensional nature of functional magnetic resonance imaging (fMRI) data and thus outperform mass-univariate tests in detecting brain–behavior associations (Busch, Conley, & Baskin-Sommers, 2025; Marek et al., 2022). Considering the research reviewed above, we hypothesized that low estrogen and high progesterone would be linked with difficulties in the neural control of approach–avoidance tendencies, and that this effect would in turn be associated with worse mood.

## Methods

### Participants

Participants were recruited between June 2023 and April 2024 via posters and flyers at the University of Barcelona. A screening survey was first sent to all who expressed interest in participating. The exclusion criteria were a history of neurological or hormonal disorders; current use of hormonal treatments, including oral contraceptives; not menstruating; a psychiatric diagnosis in the last 6 months; and MRI contraindications such as claustrophobia or large ferromagnetic implants. The total sample consisted of 116 healthy women between 18 and 35 years of age (age = 24.01 ± 4.72 [mean ± standard deviation]). Some participants were excluded from certain sets of analyses, which will be indicated where appropriate. The minimal sample size for correlation analyses (see *Brain–hormone–behavior correlations*) was *n* = 101, which falls below the preregistered target sample of *n* = 120. At a conventional two-sided threshold of *p* < .05, a sample of *n* = 101 affords a statistical power of 86% to detect correlations of *r* = .3, 71% power to detect correlations of *r* = .25, and 52% power to detect correlations of *r* = .2. These effects sizes can be, respectively, considered large, medium-to-large, and medium according to recent empirical benchmarks (Funder & Ozer, 2019).

The study was approved by the UB Ethics Committee (code IRB00003099). Participants provided informed consent prior to participation and were economically compensated afterward. The study was pre-registered on the open science framework (https://osf.io/kh2jf). All study materials will be uploaded to the OSF platform (https://osf.io/g6hb2/).

### Procedure

After recruitment, participants completed a second survey that covered demographics, the Emotion Regulation Questionnaire (Cabello, Salguero, Fernández-Berrocal, & Gross, 2013), and an ad-hoc hormonal screening questionnaire (see Supplementary Material). Participants who scored higher than 8 (out of 19) on the screening questionnaire were not invited to participate so as to exclude individuals with potentially undiagnosed endocrine disorders, such as polyendocrine metabolic ovarian syndrome (Teede et al., 2026). The Emotion Regulation Questionnaire has two scales, each indexing a different regulation strategy (reappraisal and suppression). The instrument demonstrated acceptable internal consistency in this sample (McDonald’s ω = .75).

Once at the scanner facility, participants provided informed consent and were asked to fill a tube with saliva for hormone quantification (see *Hormone level quantification* section) while reading the task instructions. Before entering the fMRI scanner, participants performed 20 practice trials of the task on a computer and were required to take off metallic items. Once the participant entered the scanner, a 5-min anatomical scan took place. Next, they completed a 240-trial AAT (20 min.) and a second 20-min task that will be reported in a separate manuscript. Stimuli were presented via fMRI-compatible goggles, which, if needed, were equipped with diopter-graded lenses to correct for the participant’s degree of refractive error (myopia or hypermetropia). After scanning, participants reported the current day of the menstrual cycle and filled out the Profile of Mood Scales or POMS (Andrade et al., 2013), which showed excellent internal consistency in this sample (McDonald’s ω =.94). The POMS was formulated to inquire about the preceding seven days, a time window during which mood might have been plausibly influenced by hormonal levels measured at the time of the experiment. Measurements were scheduled between 4 and 7 PM to limit circadian variability. The total time for the completion of the experiment was approximately 70*–*90 min per participant.

### Hormone level quantification

Before entering the scanner, participants were asked to fill a 4 mL plastic tube with saliva. Saliva production was stimulated with chewing gum, keeping the same brand and flavor constant across participants. Though testosterone and estrogen levels are affected by chewing gum mastication (van Anders, 2010), this method yields larger amounts of saliva and hastens collection. Saliva samples were stored at a temperature of -20 °C, proven not to introduce loss of components or large error variance (Toone et al., 2013). Salivary estradiol and progesterone were quantified using commercially available Enzyme-linked immunosorbent assay (ELISA) kits (Cayman Chemical, Ann Arbor, MI), following the manufacturers’ protocols. Each sample was diluted in ELISA buffer and assayed in duplicate. Briefly, standards and samples were incubated in microplate wells with tracer and antiserum for 1-2 h at room temperature, followed by washing and color development using Ellman’s reagent. Absorbance was measured at 412 nm, and hormone concentrations were interpolated using a four-parameter logistic curve. The assay range was 8.2–2,000 pg/mL for estradiol and 7.8–1,000 pg/mL for progesterone. Mean coefficients of variation across duplicates were 17% for estradiol and 12% for progesterone.

### Approach–avoidance task

A 240-trial AAT was used to measure control over automatic approach–avoidance tendencies. In half of the trials, participants were asked to approach happy faces and avoid angry ones (congruent condition), whereas, in the other half, they had to avoid happy faces and approach angry ones (incongruent condition). Pressing the ‘approach’ button made stimuli become sequentially larger, thus giving the impression of looming closer, whereas pressing the ‘avoid’ button rendered stimuli smaller, making them seemingly recede. Faces were presented for up to 2 s, with a 2-4 s jittered intertrial interval. Congruent and incongruent trials were presented in 30-trial consecutive blocks starting always with a congruent block. The start of each block was announced for 2 s with the corresponding instruction (e.g., ‘Happy = approach’ and ‘Angry = avoid’). Face stimuli were randomized within each block. Faces were extracted from the Radboud Faces Database (Langner et al., 2010). There were 30 photos of different individuals (15 women), who were displayed in two different photos with a happy or angry face.

### Neuroimaging data acquisition

Magnetic resonance imaging (MRI) scanning 3T General Electric Discovery MR750w scanner with a T2*-weighted sequence optimized for Blood Oxygen Level Dependent (BOLD) for the acquisition of neuroimaging data. A conventional T1-weighted gradient echo-planar sequence with 180 sagittal slices was used to obtain the anatomical images. The parameters for this sequence were the following: repetition time (TR)=7.7, echo time (TE)=3.5, field of view (FoV)=240, matrix=240 x 240 mm, flip angle=8°, and voxel size=1 mm isotropic. Blood-Oxygenation-Level-Dependent (BOLD) imaging was performed using a T2*-weighted gradient echo-planar sequence that included 47 axial slices per volume. The parameters for the functional sequence were the following: TR = 2.5 s, TE = 25 ms, FoV = 200 mm, matrix = 80 × 80 mm, flip angle = 90°, and voxel size = 2.5 mm isotropic. At the start of every run, five dummy scans were acquired and discarded to guarantee steady-state tissue magnetization.

### Behavioral data analysis

All analyses in this and the *Brain–hormone–behavior correlations* section were performed in RStudio version 2026.01.0 ‘Apple Blossom’ with base R version 4.5.1 ‘Great Square Root’. For the AAT, we excluded trials with reaction times below 150 ms as well as participants with error rates above 2 standard deviations from the mean, which resulted in the exclusion of six subjects (final *n*=110). We extracted mean reaction times and error rates per condition and modelled these as a function of within-subject factors movement (approach vs avoid) and emotion (angry vs happy) in repeated measures analyses of variance (rmANOVA). Significant effects (*p* < .05) were followed up with post hoc paired t-tests comparing between emotional expressions (i.e., approach angry vs approach happy and avoid angry vs avoid happy). Following previous work (Kaldewaij et al., 2019), we extracted the difference between incongruent and congruent trials (henceforth, the AAT effect) for subsequent correlation analyses.

For the POMS, we ran a principal component analysis upon observing that most POMS facets correlated substantially with each other. We retained the first principal component, on which all variables loaded moderately and which accounted for 42% of the variance in observed scores. Thus, higher scores in this component (henceforth ‘Mood’) indicate higher friendliness (*r* = .29) and vigor (*r* = .36) along with lower fatigue (*r* = −.39), anger (*r* = −.47), tension (*r* = −.42), and depression (*r* = −.46) during the last week. This analysis was performed using the prcmp function from the base R stats package.

### Neuroimaging data analysis

Neuroimaging data were preprocessed and analyzed using Statistical Parametric Mapping 12 running on MATLAB R2022b. Preprocessing entailed slice timing correction interpolated to the middle slice, realignment to the first functional volume, spatial normalization to 2.5 mm isotropic voxels in Montreal Neurological Institute (MNI) space, and smoothing with an 8 mm Gaussian kernel. Smoothing may slightly reduce the discriminability of multivoxel activity patterns by smearing informative signal variance across neighboring voxels (Misaki, Luh, & Bandettini, 2013). However, many studies report that moderate smoothing (i.e., 2-3 times the voxel size) actually improves classification accuracy (Gardumi et al., 2016; Mandelkow, de Zwart, & Duyn, 2017), especially at higher levels of signal noise (Swisher et al., 2010; Wein et al., 2025). Four further participants (final n=106) were excluded from the fMRI analyses due to excessive movement, which was determined by an average framewise displacement (FD) above 0.5 mm, and/or by having >20% volumes with FD >0.5% (Power et al., 2012).

First-level (i.e., within-participant) analyses were fit in preparation for multivoxel pattern analysis (MVPA). Each trial was defined as a single-event or ‘spike’ regressor, along with movement parameters derived from realignment. The signal was high-pass filtered with a 128 s cutoff. The resulting beta images, which index the BOLD signal variance attributable to each trial, were then fed to a multiclass support vector machine classifier with default hyperparameters as implemented in The Decoding Toolbox (Hebart, Görgen, & Haynes, 2015). Specifically, for each participant, we trained the classifier to predict which task condition was present in even trials from the activation patterns observed in odd trials. Decoding was performed using a three-voxel radius searchlight limited to each participant’s normalized gray matter mask.

At the second (i.e., between-participant) level, we performed a one-sample t-test on the resulting brain maps, which index the degree to which activation patterns around a given voxel distinguish between conditions (expressed as accuracy minus chance). The statistical threshold for whole-brain analysis was set at *p* <.001 uncorrected at the voxel level, with a cluster-level family-wise error (FWE) correction of pFWE<0.05.

Additionally, following our a priori hypotheses, we used the Wake Forest University Pickatlas (Maldjian, Laurienti, Kraft, & Burdette, 2003) to create an anatomical amygdala region-of-interest (ROI) as well as a functional lateral frontal pole (FPl) ROI. The FPl ROI was defined as two 10-mm spheres centered around the activation peaks (*x* = 32, *y* = 54, *z* = 8, and *x* = −30, *y* = 58, and *z* = 4) evoked by the incongruent > congruent contrast of the AAT across studies (Koch et al., 2018). Contrast values within these two ROIs were subject to a pFWE<.05 small-volume correction at the voxel level. For the significant ROIs identified in the FPl, striatum, and insula, we extracted each participant’s peak voxel value to be used in subsequent correlation analyses. We limited our analyses to these regions because (a) their involvement in emotional processing and regulation is clearer – and likely more direct – than for other significant clusters that emerged in the cerebellum (Pierce et al., 2022) and primary visual cortex (Liu et al., 2022), (b) because significant clusters in the latter areas were large and extended into other structures, which curtails anatomical specificity, and (c) to limit the number of multiple comparisons.

### Hormonal data analysis

We validated the hormonal measurements by comparing estradiol and progesterone levels for women in the early vs late phase of the menstrual cycle with a Welch two sample t-test, which is robust to heteroscedasticity violations. Menstrual cycle phase was defined by approximating ovulation according to each participant’s self-reported usual cycle length (day 10 for cycles<21 days, day 12 for cycles of 21–24 days, day 14 for cycles of 25–30 days, day 16 for cycles of 31–35 days, and day 18 for cycles >35 days).

### Brain–hormone–behavior associations

We computed Pearson correlation coefficients between the two ERQ scales, the Mood component extracted from POMS scores, the AAT effect for reaction times and error rates, and the FPl, insula, and striatum ROIs. Significance was set at p <.05 (uncorrected). To evaluate the robustness of significant associations, we subjected the correlation coefficients to bootstrap resampling (5,000 resamples). Therefore, associations are only flagged as significant if the 95% bootstrapped confidence interval (95% Boot. CI) does not include zero. We did not examine associations involving the progesterone-to-estradiol ratio because hormone ratios can be statistically unstable and sensitive to measurement error, which may exaggerate noise and reduce interpretability (Del Giudice & Gangestad, 2022). Furthermore, we tested for menstrual cycle effects by running Welch two-sample t-tests comparing participants in the early vs late phase on mood, task, and ROI scores.

Following our preregistered analysis plan (https://osf.io/kh2jf), we ran mediation analyses testing whether progesterone (independent variable) impacted mood (dependent variable) as a function of FPl classification accuracy (mediator). Though mediation must be interpreted with caution in cross-sectional data (Rohrer, Hünermund, Arslan, & Elson, 2022), recent experimental evidence strongly supports a causal effect of progesterone on mood (Wei et al., 2025). Note also that mediation can – and perhaps should – be tested even when the predictor of interest has no main effect on the outcome (O’Rourke & MacKinnon, 2018). As a robustness check, we ran a reversed mediation model where we swapped the roles of progesterone and FPl accuracy. The presence of an indirect effect in the main model but not the reverse one can lend support to the hypothesized mechanism (Kim et al., 2018), with the caveat that both models derive from the same covariance matrix and are thus statistically equivalent (Thoemmes, 2015). Both mediation models were specified with the lavaan package v0.6-21 (Rosseel, 2012) using a full information maximum likelihood estimator (Lee & Shi, 2021) and bootstrapping (5000 iterations), which circumvents normality violations (Pituch & Stapleton, 2008). Effects were considered significant if their 95% Boot. CI did not contain zero. We ran each model several times to ensure that the resulting estimates were consistent across resampling runs.

## Results

### Behavioral results

**Figure 1.**
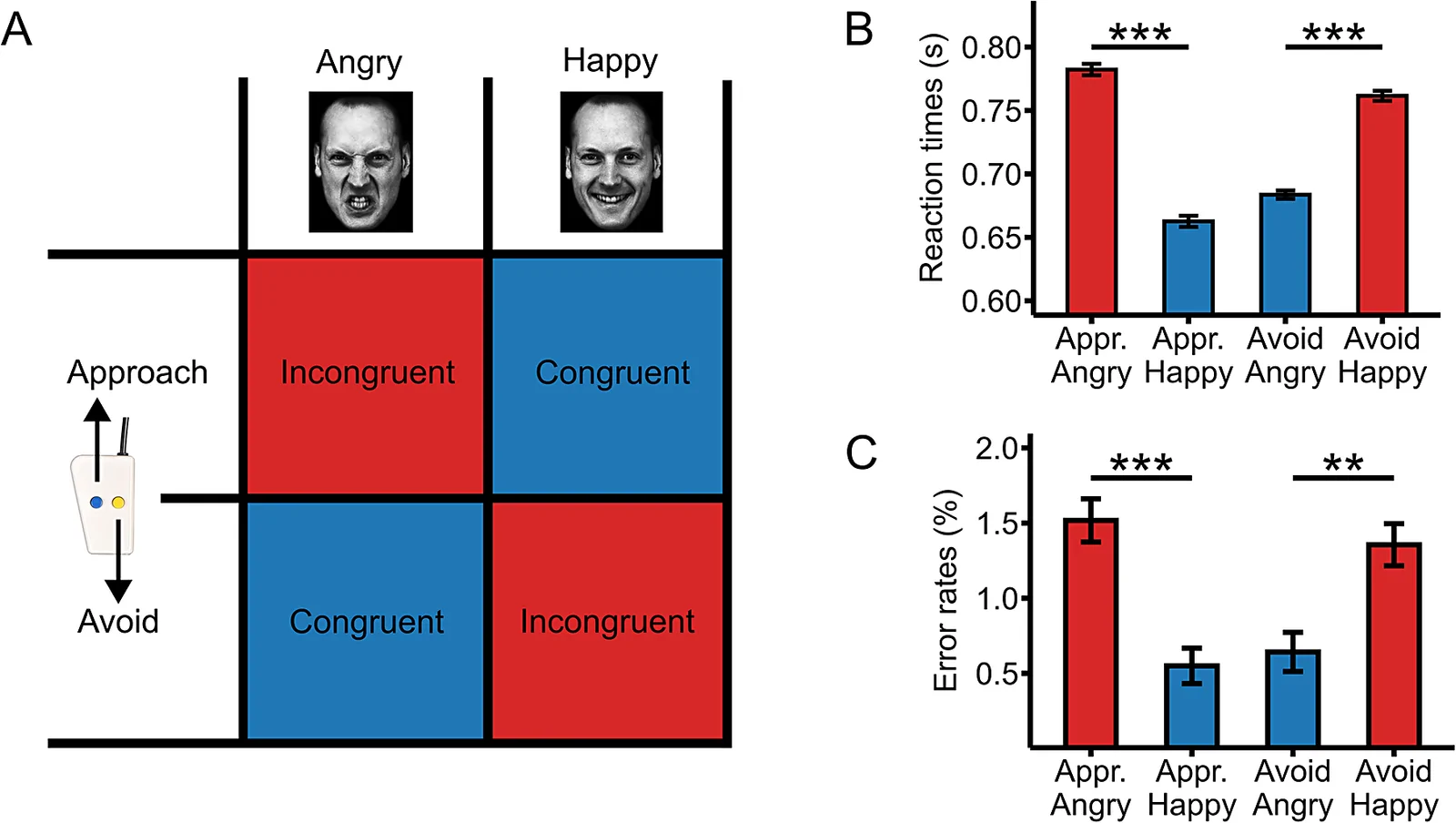
(a) Outline of the task conditions. Participants had to push a button that enlarged the image (approach) or shrunk it (avoid) depending on the emotional expression (angry or happy), resulting in four conditions, two incongruent ones (approach angry and avoid happy) and two congruent ones (approach happy and avoid angry). (b) Mean reaction times in seconds. (c) Error rates in percentage. Values are mean ± standard error of the mean (*n*=110). **p<.01, ***<*p.*001. Figure 1. long description.

For reaction times (Figure 1a), there were no differences in approach vs avoid movements (F_1,109_ = .001, *p* = .966). The main effect of emotion (F_1,109_ = 29.56, *p* < .001) was however qualified by an emotion-by-movement interaction (F_1,109_ = 29.12, *p* < .001). For error rates (Figure 1b), there was a significant interaction between emotion and movement (F_1,109_ = 19.67, *p* < .001) but not main effects of either factor alone (emotion: F_1,109_ = 1.31, *p* = .253; movement: (F_1,109_ = .07, *p* = .780). Thus, as shown in Table 1 and Figure 1, participants were generally slower and committed more errors in incongruent relative to congruent trials.

**Table 1.** Descriptive statistics for the approach–avoidance task Table 1. long description.

	RT mean	RT SE	ER mean	ER SE
Approach angry	0.782	0.014	1.517	0.221
Approach happy	0.663	0.011	0.55	0.109
Avoid angry	0.684	0.011	0.642	0.122
Avoid happy	0.762	0.013	1.356	0.225

RT, reaction times in seconds; SE, standard error of the mean; ER, error rates in percentage.

### Neuroimaging results

**Figure 2.**
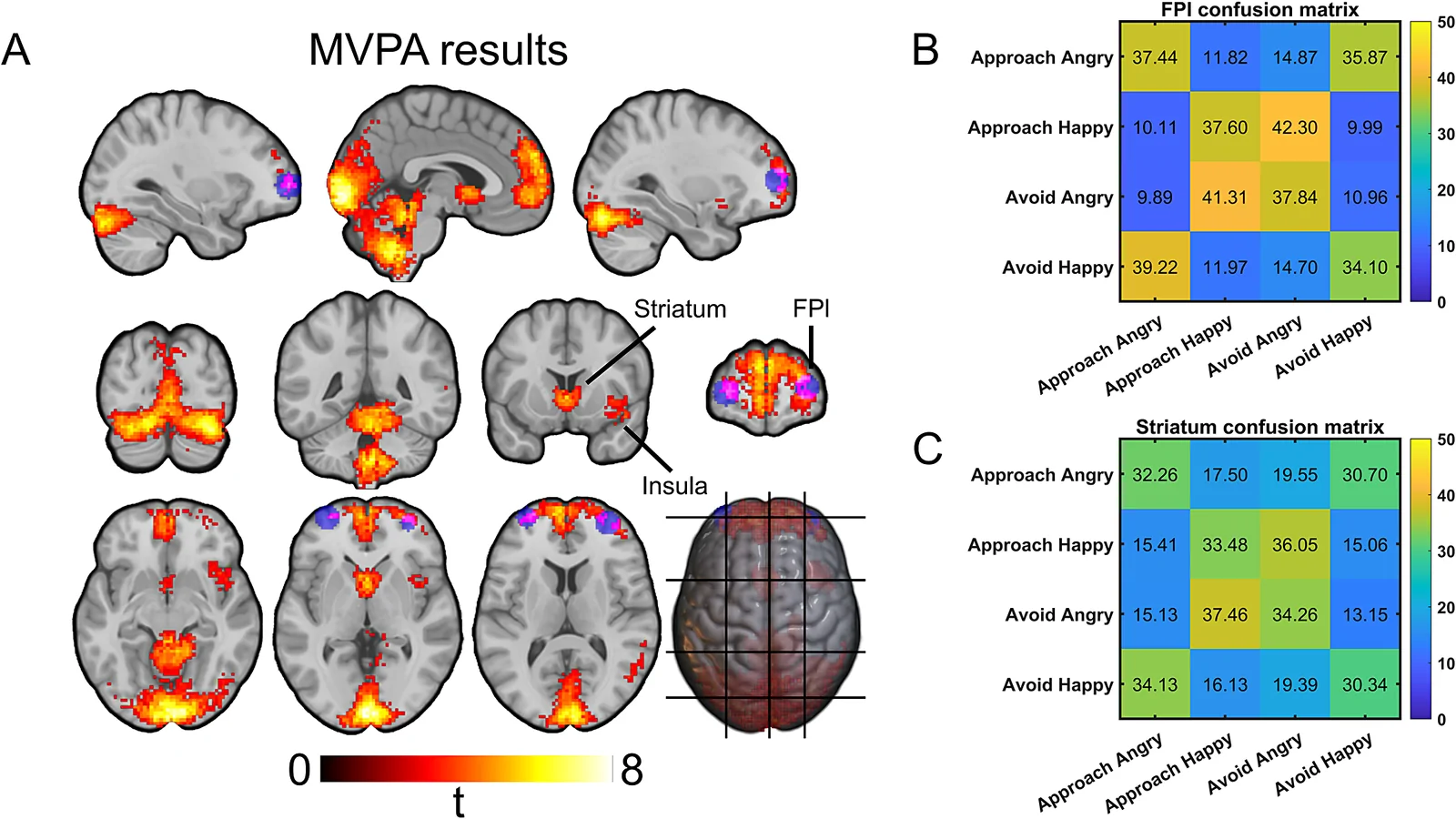
(a) Multivoxel pattern analysis (MVPA) results. Brain maps displayed at p<.005 for visualization with a FWE correction of *p*<.05 at the cluster level. Brighter colors indicate higher classification accuracy. Blue depicts the lateral frontal pole (FPl) region-of-interest (ROI). Pink denotes significant voxels within this ROI. (b) Confusion matrix for the FPl. Rows are true condition labels, and columns represent predicted labels. Chance accuracy was 25%. (c) Confusion matrix for the striatum. Figure 2. long description.

We then tested which brain areas could significantly distinguish between conditions according to their multivoxel activity patterns. This analysis revealed a large cluster (*k* = 5,756) with regional peaks in the primary visual cortex (*t* = 8.41, *x* = −2, *y* = −89.5, *z* = −2.5) and the posterior-dorsal cerebellum (*t* = 7.89, *x* = −2, *y* = −90, *z* = −2). However, as shown in Figure 2a, more ventral-anterior aspects of the cerebellum as well as the brainstem – particularly the periaqueductal gray (PAG) – also encoded task conditions above chance.

Another large cluster emerged covering most of the rostral–medial prefrontal cortex (*t* = 6.40, *x* = −7, *y* = 58, *z* = 22, *k* = 1,625). This cluster extended into our independently defined lateral frontopolar (FPl) ROI, and survived small volume correction at pFWE <.05 within this volume in both hemispheres (right: *t* = 4.69, *x* = 25.5, *y* = 55, *z* = 12.5, *k* = 35; left: *t* = 4.69, *x* = −29, *y* = 58, *z* = 5, *k* = 42).

Further, we observed above-chance decoding accuracy in the striatum, near the caudate head (*t* = 5.48, *x* = 0, *y* = 6, *z* = −2, *k* = 181). The amygdala did not significantly distinguish between conditions, even when limiting the analyses to this structure. Finally, the central insula also demonstrated above-chance condition decoding (*t* = 4.21, *x* = 43, *y* = 8, *z* = −12, *k* = 53). As shown in the confusion matrices of Figure 2b and 2c, incongruent trials (Approach Angry and Avoid Happy) could be robustly discriminated from congruent trials (Approach Happy and Avoid Angry). However, the conditions within incongruent and congruent blocks were often misclassified.

### Brain–hormone–behavior correlation results

**Figure 3.**
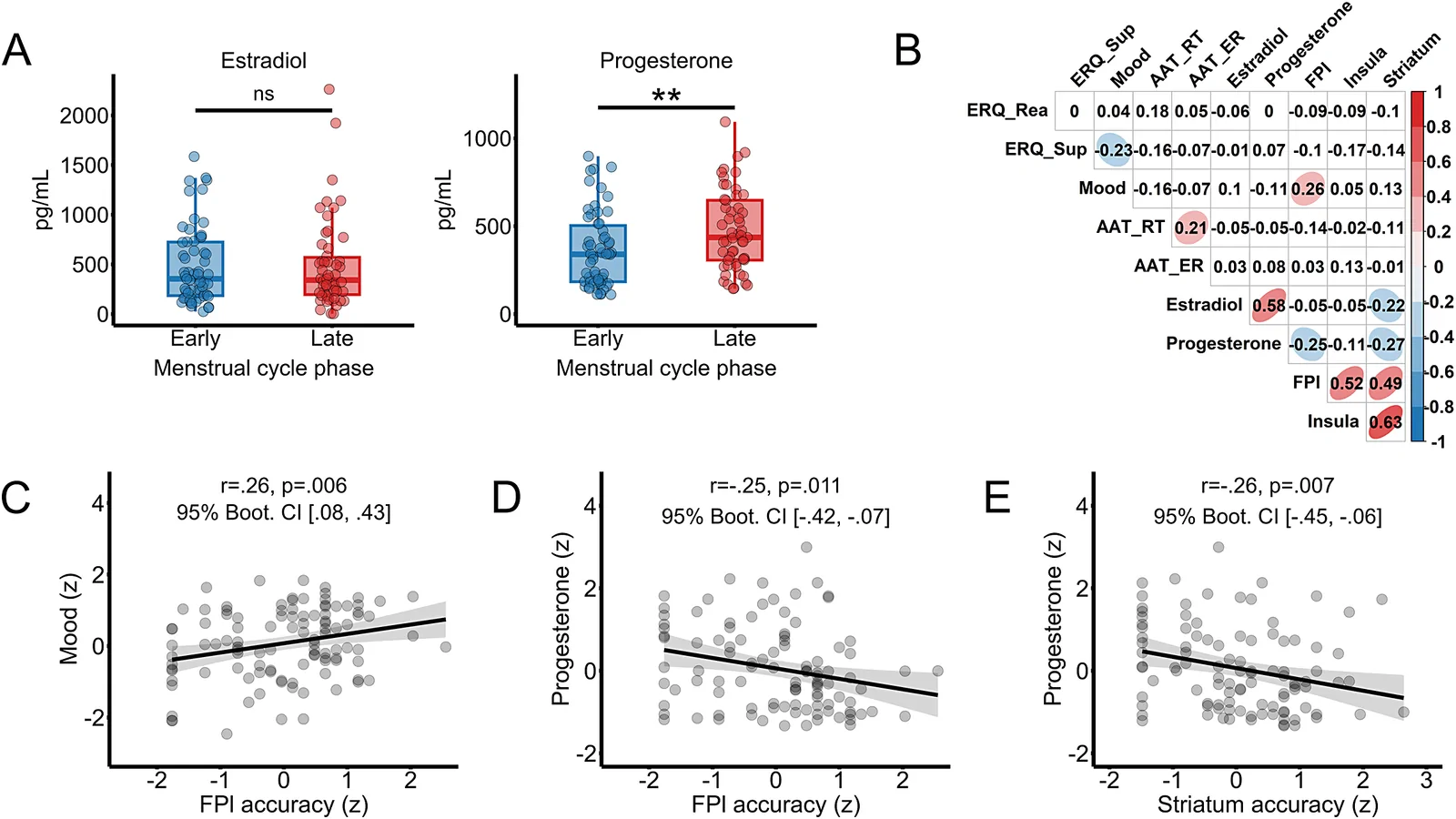
(a) Salivary estradiol (left) and progesterone (right) concentrations in the early vs late phase of the menstrual cycle. Values are mean ± standard error. **p<.01, ns: nonsignificant. (b) Correlogram displaying Pearson correlation coefficients between the main variables of interest. Colored cells mark significant associations at *p*<.05; narrower ellipses and darker hues indicate stronger associations. (c) Correlation between lateral frontal pole (FPl) classification accuracy and better mood during the past week with 95% bootstrapped confidence intervals (Boot. CIs). (d) Correlation between FPl classification accuracy lower progesterone. (e) Correlation between classification accuracy in the striatum and lower progesterone. Figure 3. long description.

We inspected for differences in saliva estradiol and progesterone concentrations as a function of menstrual cycle phase, defined relative to the approximate ovulation day expected for each participant’s habitual cycle length. Estradiol did not differ between early and late menstrual cycle stages (Figure 3a; t_104.81_ = .05, *p* = .954), but progesterone was higher in the late compared to the early phase (Figure 3b; t_106.43_ = 2.64, *p* = .009).

FPl decodability was correlated with better mood (*r* = .26, 95% Boot. CI [.08, .43], *p* = .006; Figure 3c) and lower progesterone (*r* = −.25, 95% Boot. CI [−.42, −.07], *p* = .011; Figure 3d). Progesterone was also associated with reduced classification accuracy in the striatum (*r* = −.26, 95% Boot. CI [−.45, −.06], *p* = .007; Figure 3e). Further, higher emotional suppression was linked with worse mood (*r* = −.23, 95% Boot. CI [−.39, −.06], *p* = .012). Correlations between estradiol and progesterone as well as among ROIs were high but not redundant (rs = [.49, .63]). There were no differences between participants in the early vs late phase of the menstrual cycle in any of these variables (all *t* ≤ 1.11, all *p* ≥ .266).

**Figure 4.**
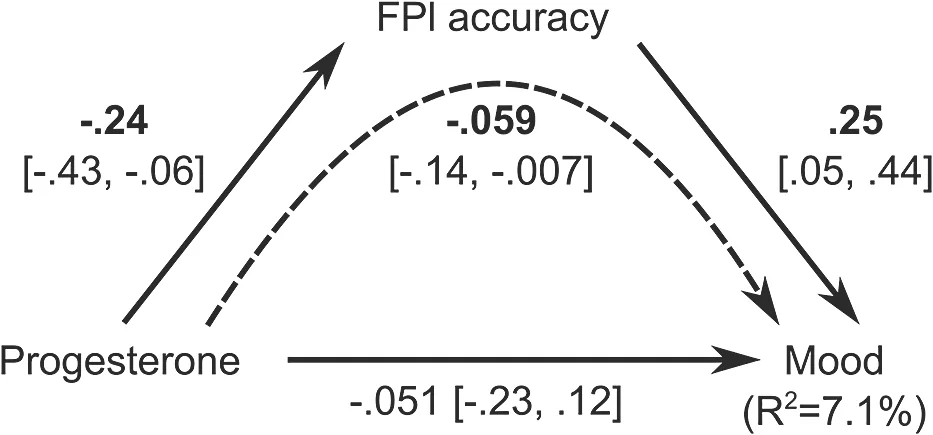
Mediation model indicating that classification accuracy in the FPl fully mediates the effect of progesterone on mood. Numbers are standardized parameter estimates along with 95% bootstrapped confidence intervals (95% Boot. CI). Solid lines depict direct effects, and the dashed line depicts the indirect effect of progesterone through FPl accuracy. Bold lettering depicts significant coefficients, i.e., those that did not contain zero in their 95% Boot. CI. R^2^: explained variance in mood.

Regarding the mediation results (Figure 4), progesterone had a small but significant indirect effect on mood (beta=.059, 95% Boot. CI [−.14, −.007]) that was fully mediated by FPl decodability. This model explained 7.1% of the observed variance in mood. In the control model, the indirect effect of FPl accuracy via progesterone was nonsignificant and substantially smaller than in the main model (beta = .012, 95% CI [−.031, 0.07]).

## Discussion

Recent years have seen a surge of research on the role of estradiol and progesterone in women’s mental health. Nevertheless, it is still uncertain how ovarian hormones modulate distributed neural patterns underlying the control of affect. In this study, we showed that mood is associated with the multivariate mapping of emotion–action contingencies in the lateral frontopolar cortex (FPl). Crucially, however, progesterone was correlated with lower task decodability in both FPl and striatum. Classification accuracy in the FPl further mediated the association between progesterone and lower mood. In addition, we observed that a host of other brain regions also contain relevant information for emotion control, including the primary visual cortex, cerebellum, PAG, striatum, and insula.

### Approach–avoidance incongruence effects on reaction times and error rates

Participants showed slower and less accurate responses in incongruent (approach angry and avoid happy) relative to congruent (approach happy and avoid angry). trials. Our results replicate the well-documented incongruence effect often observed in the AAT (Roelofs et al., 2023), which broadly concurs with other interference effects (Hedge et al., 2018). Unlike other classic paradigms such as the Stroop or flanker tasks, however, the AAT relies on automatic emotional tendencies to generate response conflict. At the same time, the AAT differs from standard emotion regulation paradigms that demand the implementation of a single strategy and thus do not require monitoring and implementing different response options (Koch et al., 2018). The AAT is therefore particularly poised to capture emotion control ability, and, indeed, both error rates (Buades-Rotger et al., 2021) and reaction times (Dapprich et al., 2021) have shown remarkable predictive validity -but see Struijs et al. (2018). Hence, replicating the established incongruence effect in the AAT attests to the success of the main experimental manipulation and lends credibility to the identified neuroendocrine correlates of emotion control. Note also that trait emotional suppression as measured with the Emotion Regulation Questionnaire was associated with worse mood, replicating another established effect (Boemo, Nieto, Vazquez, & Sanchez-Lopez, 2022) and speaking for the convergent validity of these measures.

### Neural encoding of emotion control

We observed that the FPl represents emotion–action contingencies as assessed with the AAT. The present results closely replicate those of Lapate et al. (2022), who found that action–emotion congruence could be decoded from activity patterns in the FPl (but not the amygdala) using an equivalent task. More generally, the FPl has been shown to contain state representations that aid decision-making in rapidly shifting contexts (Nassar, McGuire, Ritz, & Kable, 2019). Notably, the FPl differs strongly between human and nonhuman primates in its microstructure and connectivity (Neubert et al., 2014; Semendeferi et al., 2011), which aligns with the notion that the lateral prefrontal cortex encodes progressively more abstract information along a caudal-to-rostral gradient (Pagnotta, Riddle, & D’Esposito, 2024). All these findings, together with ours, posit the FPl as a potential target for brain stimulation in women suffering from mood dysregulation. Indeed, FPl stimulation has already demonstrated its efficacy in improving AAT performance (Bramson, den Ouden, Toni, & Roelofs, 2020) and has shown promise in alleviating social anxiety (Meijer, Bramson, Toni, & Roelofs, 2024).

The central insula as well as the striatum were also able to classify approach-avoidance contingencies. Trait perspective-taking has been associated with increased classification accuracy of emotions in the central insula (Vaccaro et al., 2022). On the other hand, there is evidence that anterior-ventral aspects of the striatum contain choice-predictive multivoxel signals during the retrieval of stimulus-outcome associations (Guidotti, Tosoni, & Sestieri, 2020). Multivariate activity patterns in the same region have also been shown to encode social rewards (Wake & Izuma, 2017) and competitive prowess, strategically facilitating antagonistic behavior (Buades-Rotger et al., 2021). This evidence suggests, along with the present findings, that the insula supports the encoding of emotional information, whereas the striatum may be involved in transforming these social–emotional signals into adaptive motor responses.

The cerebellum could also differentiate between congruent and incongruent emotion–action links. This effect encompassed lobules V and VI as well as Crus I, the cerebellum subregions that most robustly encode emotional expressions across studies (Pierce et al., 2022). A recent investigation has linked cerebellar–FPl connectivity with the processing of negative emotions as well as self-reported anxiety (Wong et al., 2025). Of note, the cerebellum cluster extended into the PAG, a region that has been involved in threat detection and in initiating subsequent fight-or-flight responses (Hulsman et al., 2024; Lefler, Campagner, & Branco, 2020). Therefore, we speculate that cerebellum-dependent motor plans are relayed downstream to the PAG in order to jointly orchestrate affect–appropriate responses. The identification of correct emotion–response contingencies might have been in turn supported by the primary visual cortex, which, in line with previous studies (Dobs, Schultz, Bülthoff, & Gardner, 2018; Muukkonen & Salmela, 2022), significantly distinguished between emotional expressions.

### Progesterone-dependent modulation of multivoxel activity patterns

Critically, we observed that progesterone was linked with diminished task decodability in the FPl and striatum. Moreover, mediation analyses showed that FPl decodability fully accounts for the indirect effect of progesterone on mood. Reverse mediation of FPl accuracy through progesterone was weaker and not significant, consistent with – but not demonstrating – the hypothesized direction. Progesterone receptors are particularly expressed in the hippocampus but are present throughout the brain (Bencker et al., 2025). Our results are broadly in line with findings of increased neurofunctional variability as a function of endogenous progesterone (Avila-Varela et al., 2024). More diffuse neural activation patterns might hence impair successful emotional processing, contributing to the emotional lability that many women report in the late stages of the menstrual cycle. Yet, such a phenomenon could also promote ‘luteal analgesia’, wherein women show lower neural and self-reported pain sensitivity in a high-progesterone state (Vincent et al., 2018). Therefore, progesterone seems to induce a relative affective blunting that may nonetheless compromise effortful emotion control. This is consistent with the finding that oral contraceptives, whose main active ingredient is progestin (a synthetic progesterone analog), suppress threat avoidance but facilitate approach behavior toward positive stimuli after stress (Thurley et al., 2024). In sum, our findings add to the notion that progesterone can act as a ‘double-edged sword’ (Sundström-Poromaa et al., 2020) in that it may generally have an adverse effect on mood while its desensitizing properties can be adaptive in certain contexts.

Stiernman et al. (2025) reported greater limbic and paralimbic reactivity to emotional stimuli in women with premenstrual dysphoria but did not find a direct link between this effect and progesterone-derived neuroactive steroids. Our study suggests that, with a larger sample size and individualized multivariate measures, it may be possible to detect these associations – even if measurement variability renders them elusive or imprecise. Still, there were a number of null findings that warrant attention. Most notably, neither estradiol nor progesterone correlated with reaction times or error rates in the AAT. This might be due to the low reliability of individual task scores (Hedge, Powell, & Sumner, 2018), a problem that future studies might alleviate using generative modeling (Haines et al., 2025). Unreliability also affects fMRI estimates (Elliott et al., 2020), but this issue was partially mitigated with cross-validated multivariate methods (Busch et al., 2025; Marek et al., 2022). The null association between estradiol and mood was also unexpected, considering the positive correlations reported in recent, well-powered studies (Jansen et al., 2023; Pletzer & Noachtar, 2023). Estradiol estimates were 5% more variable across aliquots than those of progesterone. Concordantly, salivary estradiol fares worse than progesterone as a menstrual cycle marker (Arslan et al., 2023), a finding that we also replicate here. In addition, progesterone tracks neurostructural changes across typical menstrual cycles more closely than estradiol (Heller et al., 2025). Measurement noise might have hence precluded the detection of significant associations with estradiol. The null effects of menstrual cycle phase (early vs late) on most variables are also worth mentioning, as they add to the growing evidence documenting no systematic differences across the menstrual cycle in several measures of affective and cognitive function (Pletzer et al., 2019; Pletzer & Noachtar, 2023; Rafiee et al., 2023). Hence, notwithstanding the limitations outlined in the following section, emotional state seems to be more strongly related to neuroendocrine parameters than to the current menstrual cycle phase per se.

### Limitations and future directions

A few notes of caution should be further considered when interpreting our results. First, we only took one saliva sample per participant, and measurements took place at different stages of the menstrual cycle, as in previous studies (Chung et al., 2019; Sharma et al., 2021; Wang et al., 2020). Therefore, our data cannot cleanly disentangle trait and state estimates. Nevertheless, we speculate that the neuroendocrine mechanisms identified in the present study reflect transient affective-cognitive states rather than stable features. This notion is supported by the finding that progesterone was associated with mood but not with trait emotion regulation. Furthermore, recent evidence has linked progesterone fluctuations to *changes* in Premenstrual Dysphoric Disorder (PMDD) symptoms (Ko et al., 2024; Wei et al., 2025) rather than their overall severity (Ko et al., 2024). Our assumption must be however substantiated by longitudinal studies with denser sampling (Pritschet, Taylor, Santander, & Jacobs, 2021). Second, and relatedly, the observed hormonal concentrations must be considered imprecise due to (a) the limited validity of salivary estradiol for cycle-phase inference (Arslan et al., 2023), (b) evidence that chewing gum alters steroid concentrations (Schultheiss, 2013; van Anders, 2010), and (c) the relatively high coefficients of variation observed in our assays. These factors likely introduce noise rather than systematic bias, which would pull associations toward the null – as may have also been the case for reaction times and error rates. Menstrual cycle phase estimation was coarse as well, since it was based exclusively on self-reports. Indeed, there is substantial variability in menstrual cycle length (especially in the follicular phase) even in relatively homogeneous cohorts of healthy young women (Bull et al., 2019; Henry, Shirin, Goshtasebi, & Prior, 2024). Third, our study was underpowered (52%) to detect associations of *r*=.2, but we still retained 71% power to detect associations of approximately the observed magnitude (*r*=.25). Correlations of this size could be interpreted as ‘small’ according to Cohen’s classic but admittedly arbitrary guidelines (Correll, Mellinger, McClelland, & Judd, 2020). However, typical effect sizes in psychology using adequately powered samples hover around *r*=.2, so that effect sizes of *r*=.1 can be deemed small, and those of *r*=.3 or greater can be interpreted as large (Funder & Ozer, 2019). Hence, pending replication, the effects reported here can be considered medium-to-large according to modern, realistic standards. Even so, these correlations explain a small percentage of variance in mood, and the multivariate mediation model only accounted for a modest 7.1%. As mentioned above, improved measurement models (Haines, Sullivan-Toole, & Olino, 2023), intensive longitudinal sampling (Pritschet et al., 2021), data sharing (Heller et al., 2024), and sophisticated statistical methods (Rutledge, Chekroud, & Huys, 2019) are necessary to address the complex neuroendocrinology of affect and its regulation.

## Conclusion

In this study, we investigated whether ovarian hormones can influence multivariate representations of emotion control. Incongruent trials (e.g., approaching angry faces) elicited longer reaction times and higher error rates, dovetailing previous studies. Incongruent and congruent trials could be significantly decoded from multivariate activation patterns in the FPl, striatum, insula, early visual areas, and the cerebellum. Crucially, we observed that lower frontopolar decodability of emotion–action contingencies is a progesterone-sensitive correlate of mood variation. In sum, our study provides novel and precise insights into the neuroendocrine modulation of affect in healthy young women.

## Supporting information

10.1017/S0033291726105315.sm001 Buades-Rotger et al. supplementary material Buades-Rotger et al. supplementary material

## Data Availability

Analysis scripts, behavioral data, and summarized neuroimaging data are publicly available on an Open Science Foundation (OSF) repository (https://osf.io/g6hb2/). Raw and preprocessed neuroimaging data can be provided upon reasonable request.

## Supplementary material

The supplementary material for this article can be found at http://doi.org/10.1017/S0033291726105315.

## Long descriptions

### Table 1. Long description

The table consists of five columns and five rows. The header row contains the following labels: R T mean, R T S E, E R mean, and E R S E. The subsequent rows detail the results for four conditions:

* Row 1: Approach angry. R T mean 0.782, R T S E 0.014, E R mean 1.517, E R S E 0.221.

* Row 2: Approach happy. R T mean 0.663, R T S E 0.011, E R mean 0.55, E R S E 0.109.

* Row 3: Avoid angry. R T mean 0.684, R T S E 0.011, E R mean 0.642, E R S E 0.122.

* Row 4: Avoid happy. R T mean 0.762, R T S E 0.013, E R mean 1.356, E R S E 0.225.

Note: R T refers to reaction times in seconds, S E refers to standard error of the mean, and E R refers to error rates in percentage.

Navigate back to Table 1..

### Figure 1. Long description

Panel A is a two-by-two matrix. The columns are labeled Angry and Happy with corresponding facial photos. The rows are labeled Approach and Avoid with an illustration of a joystick. The matrix cells are: top-left red Incongruent (Approach Angry), top-right blue Congruent (Approach Happy), bottom-left blue Congruent (Avoid Angry), and bottom-right red Incongruent (Avoid Happy).

Panel B is a bar graph of Reaction times in seconds. The y-axis ranges from 0.60 to 0.80. Four bars represent the conditions: Appr. Angry (red, approx. 0.78), Appr. Happy (blue, approx. 0.66), Avoid Angry (blue, approx. 0.68), and Avoid Happy (red, approx. 0.76). Triple asterisks indicate significant differences between the first two and last two bars.

Panel C is a bar graph of Error rates in percentage. The y-axis ranges from 0 to 2.0. The bars are: Appr. Angry (red, approx. 1.5), Appr. Happy (blue, approx. 0.5), Avoid Angry (blue, approx. 0.6), and Avoid Happy (red, approx. 1.3). Triple asterisks mark the first pair and double asterisks mark the second pair, indicating statistical significance.

Navigate back to Figure 1..

### Figure 2. Long description

Panel A, titled M V P A results, contains eleven brain slices and one 3D rendering. The slices are arranged in three rows. The top row shows three sagittal views with heat map activations in the frontal and occipital regions. The middle row shows four coronal views, with labels pointing to the Striatum, Insula, and F P l. The bottom row shows three axial views and a top-down 3D view with a grid overlay. A color scale at the bottom indicates t-values from 0 (black) to 8 (white-yellow). Blue regions represent the F P l region of interest, while pink voxels indicate significant classification within that area.

Panel B is the F P l confusion matrix. The y-axis (true labels) and x-axis (predicted labels) both list: Approach Angry, Approach Happy, Avoid Angry, and Avoid Happy. The diagonal values from top-left to bottom-right are 37.44, 37.60, 37.84, and 34.10. High off-diagonal values include 35.87 (True Approach Angry predicted as Avoid Happy) and 42.30 (True Approach Happy predicted as Avoid Angry).

Panel C is the Striatum confusion matrix with the same axes. The diagonal values are 32.26, 33.48, 34.26, and 30.34. Notable off-diagonal values include 30.70 (True Approach Angry predicted as Avoid Happy) and 37.46 (True Avoid Angry predicted as Approach Happy). Both matrices use a color scale from 0 (dark blue) to 50 (yellow).

Navigate back to Figure 2..

### Figure 3. Long description

Panel A contains two box plots with individual data points overlaid. The left plot shows Estradiol levels in p g / m L for Early and Late phases, with no significant difference. The right plot shows Progesterone levels, which significantly increase from the Early phase (mean around 400) to the Late phase (mean around 500), marked with two asterisks.

Panel B is a triangular correlogram matrix. The vertical axis lists E R Q _ Rea, E R Q _ Sup, Mood, A A T _ R T, A A T _ E R, Estradiol, Progesterone, F P l, and Insula. The horizontal axis lists the same variables from Mood to Striatum. Significant correlations are highlighted with ellipses: a positive red ellipse for Estradiol and Progesterone (0.58), F P l and Insula (0.52), F P l and Striatum (0.49), and Insula and Striatum (0.63). Negative blue ellipses appear for E R Q _ Sup and Mood (-0.23) and Progesterone and Striatum (-0.27).

Panel C is a scatter plot showing a positive linear correlation between F P l accuracy (z) on the x-axis and Mood (z) on the y-axis (r equals .26, p equals .006).

Panel D is a scatter plot showing a negative linear correlation between F P l accuracy (z) on the x-axis and Progesterone (z) on the y-axis (r equals minus .25, p equals .011).

Panel E is a scatter plot showing a negative linear correlation between Striatum accuracy (z) on the x-axis and Progesterone (z) on the y-axis (r equals minus .26, p equals .007). All scatter plots include a regression line and a shaded 95 percent Boot. C I area.

Navigate back to Figure 3..
