# Supplementary material for: Endogenous progesterone blurs frontostriatal representations of emotion control in healthy young women
Source: Psychol Med. 2026 Sep 3;56:e263. doi: 10.1017/S0033291726105315 (PMC13735840; doi:10.1017/S0033291726105315)
Supplement: Supplementary file 1 [file S0033291726105315sup001.pdf]

Table S1. Descriptive statistics for questionnaire scores and hormonal concentrations

**Variable**	**Mean**	**SD**
ERQ_Suppression	2.439	0.962
ERQ_Reappraisal	3.800	0.597
POMS_Anger	2.125	0.794
POMS_Fatigue	2.691	0.987
POMS_Vigor	2.936	0.839
POMS_Friendliness	3.945	0.734
POMS_Tension	2.476	0.998
POMS_Depression	1.822	0.729
Estradiol (pg/mL)	484.621	413.568
Progesterone (pg/mL)	413.202	227.229

ERQ: Emotion Regulation Questionnaire, POMS: Profile of Mood Scales, SD: Standard Deviation. Questionnaire scores ranged from 1 to 5, hormonal concentrations ranged from 8.2–2,000 pg/mL for estradiol and 7.8–1,000 pg/mL for progesterone.

Endocrinologic screening questionnaire

Responses were scored as indicated by the numbers within parentheses.

1. **Length of the menstrual cycle in days**
   a) <21 (2)
   b) 21–24 (1)
   c) 25–30 (0)
   d) 31–35 (1)
   e) >35 (2)

2. **Changes in the menstrual cycle**
   a) Irregular periods (1)
   b) Missed periods (1)
   c) I don't usually experience changes (0)

3. **Changes in body hair**
   a) Increased hair growth (1)
   b) Hair loss (1)
   c) None of the above (0)

4. **Weight changes**
   a) Unexplained weight gain (1)
   b) Unexplained weight loss (1)
   c) None of the above (0)

5. **Skin changes**
   a) Dryness (1)
   b) Excessive oiliness (1)
   c) None of the above (0)

6. **Changes in energy and fatigue**
   a) Unexplained fatigue (1)
   b) Lack of energy (1)
   c) None of the above (0)

7. **Mood changes**
   a) Increased anxiety (1)
   b) Depression (1)
   c) Aggression (1)
   d) Irritability (1)
   e) None of the above (0)

8. **Changes in body temperature**
   a) Sudden hot flashes (1)
   b) Night sweats (1)
   c) Cold intolerance (1)
   d) None of the above (0)

9. **Breast changes**
   a) Breast tenderness (1)
   b) Discharge / secretion (1)
   c) None of the above (0)

10. **Do you take birth control pills?**
    a) Yes (excluded)
    b) No (0)

11. **How many times have you used emergency contraception (morning-after pill)?**
    (1 per occasion)
